# Identification of urinary metabolites that correlate with clinical improvements in children with autism treated with sulforaphane from broccoli

**DOI:** 10.1186/s13229-018-0218-4

**Published:** 2018-05-30

**Authors:** Stephen Bent, Brittany Lawton, Tracy Warren, Felicia Widjaja, Katherine Dang, Jed W. Fahey, Brian Cornblatt, Jason M. Kinchen, Kevin Delucchi, Robert L. Hendren

**Affiliations:** 1 Department of Psychiatry, University of California, San Francisco, 401 Parnassus, LP-119, San Francisco, CA 94143 USA; 2 Department of Epidemiology and Biostatistics, University of California, San Francisco, 401 Parnassus, LP-119, San Francisco, CA 94143 USA; 3 Departments of Medicine, Pharmacology and Molecular Sciences, International Health, and Cullman Chemoprotection Center, Johns Hopkins University, 855 N. Wolfe St. Ste. 625, Baltimore, MD 21205 USA; 4 Nutramax Laboratories Consumer Care, Inc, 2208 Lakeside Blvd, Edgewood, MD 21040 USA; 5 Metabolon, Inc, 617 Davis Dr. Suite 400, Durham, NC 27713 USA; 6 Department of Medicine, UCSF, SFVAMC, 111-A1, 4150 Clement St, San Francisco, CA 94121 USA

**Keywords:** Autism, Metabolomics, Antioxidant, Biomarker

## Abstract

**Background:**

Children with autism spectrum disorder (ASD) have urinary metabolites suggesting impairments in several pathways, including oxidative stress, inflammation, mitochondrial dysfunction, and gut microbiome alterations. Sulforaphane, a supplement with indirect antioxidant effects that are derived from broccoli sprouts and seeds, was recently shown to lead to improvements in behavior and social responsiveness in children with ASD. We conducted the current open-label study to determine if we could identify changes in urinary metabolites that were associated with clinical improvements with the goal of identifying a potential mechanism of action.

**Methods:**

Children and young adults enrolled in a school for children with ASD and related neurodevelopmental disorders were recruited to participate in a 12-week, open-label study of sulforaphane. Fasting urinary metabolites and measures of behavior (Aberrant Behavior Checklist—ABC) and social responsiveness (Social Responsiveness Scale—SRS) were measured at baseline and at the end of the study. Pearson’s correlation coefficient was calculated for the pre- to post-intervention change in each of the two clinical scales (ABS and SRS) versus the change in each metabolite.

**Results:**

Fifteen children completed the 12-week study. Mean scores on both symptom measures showed improvements (decreases) over the study period, but only the change in the SRS was significant. The ABC improved − 7.1 points (95% CI − 17.4 to 3.2), and the SRS improved − 9.7 points (95% CI − 18.7 to − 0.8). We identified 77 urinary metabolites that were correlated with changes in symptoms, and they clustered into pathways of oxidative stress, amino acid/gut microbiome, neurotransmitters, hormones, and sphingomyelin metabolism.

**Conclusions:**

Urinary metabolomics analysis is a useful tool to identify pathways that may be involved in the mechanism of action of treatments targeting abnormal physiology in ASD.

**Trial registration:**

This study was prospectively registered at clinicaltrials.gov (NCT02654743) on January 11, 2016.

## Background

Persons with autism spectrum disorder (ASD) have many physiological abnormalities compared to typically developing children. These abnormalities cluster into four areas: oxidative stress, inflammation/immune dysregulation, mitochondrial dysfunction, and environmental toxicant exposure [1]. Prior studies examining physiological abnormalities have examined biomarkers for one particular metabolic pathway (such as cytokines [2], oxidative stress [3], or mitochondrial dysfunction [4]), and a variety of others has been proposed [5]. However, recent studies have used metabolomics—the study of all metabolites present in a cell, organism, tissue, or sample (e.g., blood, urine, saliva, feces)—to more broadly examine differences in multiple, concurrent physiological mechanisms in children with ASD. These metabolomics studies comparing children with ASD to typical controls have identified many physiological abnormalities that also cluster into similar categories of dysfunctional redox status, inflammatory status, and mitochondrial function as well as metabolomic signals that suggest abnormalities in the gut microbiome (the microbial population of an individual’s gastrointestinal tract, which is increasingly recognized as an important contributor to health and disease) [6–16]. Together, all of these studies strongly suggest that persons with autism have abnormal physiology that may be intermittent or continuous.

An exciting therapeutic potential in ASD involves the treatment of these identified physiological abnormalities when they are active. Correction of these abnormalities may improve behavior, symptoms, and quality of life in ASD. Preliminary evidence from intervention trials supports this possibility. For instance, children with autism who were treated with the antioxidant *N*-acetylcysteine (NAC) for 12 weeks had significant improvements in irritability compared to children treated with placebo [17]. In a recent randomized controlled trial of methylcobalamin (methyl B12), children with ASD who were treated with this methyl donor had significant improvements in the Aberrant Behavior Checklist (ABC) compared to those treated with placebo, and improvements were correlated with physiological measures that indicated improved antioxidant status [18].

A previous, small, randomized controlled trial examined the efficacy of sulforaphane for the treatment of children with ASD [19]. Sulforaphane is an indirect antioxidant from broccoli sprout and seed extracts [20]. Sulforaphane has many physiological effects, including upregulation of cytoprotective enzymes and increases in detoxification and excretion of highly reactive and potentially damaging toxicants and free radicals. During the 18-week trial, children receiving oral sulforaphane demonstrated marked improvements in both the ABC and the SRS [19]. Further, the observed benefits disappeared and returned to baseline when the treatment was stopped. However, the above study did not examine the physiological changes in children with ASD undergoing treatment.

We therefore sought to examine changes in physiological markers that may underlie beneficial treatment effects from sulforaphane by analyzing changes in urinary metabolites. In an open-label clinical trial of 15 school-age children with ASD, we performed metabolomics analyses of urine before and after treatment to determine if there are changes in the low molecular weight products of metabolism associated with treatment response, which might explain the mechanism of action of this phytochemical.

## Methods

### Participants

The study was approved by the Committee on Human Research at the University of California, San Francisco (UCSF) on November 5, 2015, and the trial was registered at clinicaltrials.gov (NCT02654743) on January 11, 2016, prior to performing any study activities. Participants completed the study on May 9, 2016, and metabolomics analyses were completed in September 2016.

Prior to initiating the study, the UCSF investigators developed a relationship with a local, non-public school (Oak Hill School, San Anselmo, CA) that specializes in the education of children and young adults with autism and related neurodevelopmental disorders (ages 5–22, grades K-12). This unique academic-school-parent partnership was created with the goal of improving overall care and communication between caregivers, clinical providers, and teachers. All children/families attending the school (*n* = 35 at study initiation) were invited to participate in the study through e-mail, informational flyers, and an evening informational session.

Children and young adults were eligible to participate if they were enrolled in the school, had a formal diagnosis of autism, reported no prior use of sulforaphane-containing supplements, were willing to hold other treatments constant for the 12-week study period, had no major medical problems other than ASD, were willing to provide urine samples, and parents were willing to complete online surveys at specified intervals. ASD was defined as being present if the child had a diagnosis from a medical professional trained to diagnose autism or if the student was determined by school staff and the study psychiatrist to meet *Diagnostic and Statistical Manual of Mental Disorders*, Fourth Edition (*DSM–IV*) criteria for ASD. Informed consent was obtained from the parent/caregiver of all study participants.

### Intervention

The study was open-label, and all clinicians, parents, and teachers were aware of the treatment initiation and duration of 12 weeks. All enrolled children were provided weight-based dosing of sulforaphane (~ 2.5 μmol glucoraphanin (GR)/lb). Avmacol^®^, a sulforaphane-producing dietary supplement, was provided at no cost by Nutramax Laboratories Consumer Care, Inc. (Edgewood, MD). Each tablet provides a proprietary broccoli seed and broccoli sprout blend (ERS92®) delivering GR and active myrosinase enzyme and was calculated to deliver at least the same dose of sulforaphane as in the prior randomized controlled trial in autism [19, 21]. In the prior randomized controlled trial, participants were provided pure sulforaphane in contrast to the current study, where sulforaphane was delivered as its precursor (GR) along with a conversion enzyme, myrosinase, which converts GR to sulforaphane in the body (providing the advantage of greater stability and a longer shelf life). Prior bioavailability studies suggest that the GR plus myrosinase is an excellent and efficient delivery method, but there is variability from person to person and the prior bioavailability studies were conducted in adults [21]. At present, there have been no dose-efficacy trials, so it is unknown what an “optimal” dose might be. Dosing was adjusted in weight categories as follows: 32–41 kg (6 tablets = 222 μmol GR/day), 41–50 kg (7 tablets = 259 μmol GR/day), 50–59 kg (8 tablets = 296 μmol GR/day), 59–68 kg (9 tablets = 333 μmol GR/day), 68–77 kg (10 tablets = 370 μmol GR/day), 77–86 kg (12 tablets = 444 μmol GR/day), 86–95 kg (13 tablets = 481 μmol GR/day), and 95–105 kg (15 tablets = 555 μmol GR/day). Caregivers were advised to administer the tablets once a day in the morning. A simple grinding device (www.carex.com/item/70071/Ultra-Pill-Crusher) was provided to all families so that tablets could be ground and mixed into cold food (yogurt, applesauce, fruit juice or shakes, etc.), and most families provided tablets in this manner. All children completed baseline measures (described below) and provided urine samples during the same 2-week screening window, and then all children commenced treatment on the same day (January 21, 2016) (batch enrollment) and finished treatment on the same day (April 21, 2016).

### Objectives and outcomes

The primary goal of the study, defined a priori, was to determine if any observed changes in symptoms of ASD were correlated with changes in urinary metabolites. We hypothesized that sulforaphane treatment would lead to changes in markers of oxidative stress (or other physiological abnormalities) and that those changes would correlate with clinical improvements.

The two primary outcome measures were changes in behavior as measured by the Aberrant Behavior Checklist (ABC) and changes in social function as measured by the Social Responsiveness Scale (SRS); both scales are commonly used outcome measures in clinical trials of interventions in ASD. Parents/caregivers were asked to complete both measures at baseline, 4 weeks, and 12 weeks using an online and secure platform.

Urine was collected once during the 2-week window before treatment initiation and again at the end of the 12-week study period. Parents/caregivers were provided a sterile urine cup and were asked to collect a first morning urine (fasting since dinner the day before) and bring it immediately to their clinic visit, which was also arranged in the morning. Parents/caregivers were asked if the child had a medical illness at the time of urine collection, and no children had a current medical illness. Urine was immediately stored in 2-mL aliquots at − 80 °C. During the screening visit, children had a brief physical examination including height and weight to guide the proper dosing. Sulforaphane study tablets were provided at the screening visit along with dosing instructions. Parents were required to complete the intake forms online before the study coordinator contacted them to inform them to start taking the study supplement on January 21, 2016.

### Safety assessments

Parents/caregivers and teachers were advised to report any concerns about a new medical problem immediately to the study investigators, who were available at all times to receive reports of possible adverse effects. At the 4- and 12-week online questionnaires, parents/caregivers were asked to report any new medical problems or concerns for possible side effects.

### Metabolomic analyses

All urine samples were sent on dry ice in one batch to Metabolon (Morrisville, NC). Metabolomics analysis was conducted at Metabolon as previously described [22]. Briefly, samples were subjected to methanol extraction, then split into five aliquots for analysis by ultrahigh performance/mass spectrometry in the positive (two methods), negative or polar ion modes. Metabolites were identified by automated comparison of ion features to a reference library of chemical standards followed by visual inspection for quality control [23]. For statistical analyses and data display, any missing values were assumed to be below the limits of detection; these values were imputed with the compound minimum (minimum value imputation). Data was then normalized by measured osmolality, which is necessary to reduce the variability in metabolomics analyses due to differing urine concentrations [24]. A metabolic pathway for a given metabolite was assigned based on prior designations in the literature combined with experience from prior datasets at Metabolon.

### Statistical methods

Summary statistics were used to describe the variables. Change in both the clinical variables and the metabolites was computed as post-test minus pre-test. Pearson’s correlation coefficient was estimated and tested to index the association between the change in each of the two clinical scales and the change in each metabolite. Given the early-stage nature of the research, we did not adjust for multiple comparisons because we felt it was more important to risk a type II error than to miss a potentially important signal by being overly conservative, as has been suggested by prior authors [25].

We defined, a priori, that a correlation cutoff of an absolute value of ≥ 0.6 was of potential clinical relevance, because, given our sample size of 15, we would have approximately 80% power to detect a correlation of that size and such a correlation accounts for roughly one third of the variance. Others have suggested that correlations with an absolute value of ≥ 0.6 indicate a moderate or higher correlation [26] (and are hence of greatest interest in pointing to a mechanism of action).

We also examined the number of participants who had a clinical response, defined a priori as an improvement of four or more points in the ABC. We compared the pre-post changes in the ABC and SRS in the responder and non-responder groups using the Student’s *t* test.

## Results

Twenty-one of 35 students who were enrolled in the school completed the informed consent process for the study. Six families dropped out prior to completing any post-baseline information for the following reasons: two subjects withdrew consent prior to starting for unstated reasons; two subjects did not like the taste of the supplement; two parents filled out no surveys. The characteristics of the 15 children who provided follow-up information are shown in Table 1. All children had a diagnosis of ASD, 80% were male, the mean age was 14.8, and there were no known genetic conditions among study participants. Their baseline ABC hyperactivity and irritability scores (Table 2) are similar to those in the prior, seminal study of risperidone for behavioral problems in children with autism and “serious behavioral disturbances,” where baseline hyperactivity and irritability scores were 31.8 (vs. 29.4 in the current study) and 26.2 (vs. 25.0 in the current study), respectively [27].

**Table 1:** Characteristics of enrolled participants

Category	Characteristic	Subjects (*N* = 15)	
		%	*N*
Gender	Male	80	12
	Female	20	3
Ethnicity	White	60	9
	Asian/Pacific Islander	7	1
	No response	33	5
Age	7–10	13	2
	11–14	47	7
	15–21	40	6
	Mean age		14.7
Primary diagnosis	Autism spectrum disorder	100	15
Comorbidities	Intellectual disability	40	6
	Language disorder	27	4
	ADHD	19	3
	Pica	7	1
	Global Development Delay	7	1
	Learning disability	7	1
Current meds	Sertraline	13	2
	Lurasidone	7	1
	Risperidone	13	2
	Birth control	13	2
	Zonisamide	7	1

*ADHD* attention deficit hyperactivity disorder

**Table 2:** Change in outcome scores over the 12-week study period

Outcome measure	Adjusted mean scores (95% CI)			Change from baseline (95% CI)			
	Baseline	1 month	3 months	1 month	*p*	3 months	*p*
Aberrant behavior checklist total score	103.9 (72.0 to 135.9)	94.2 (63.8 to 124.6)	96.9 (65.7 to 128.0)	− 9.7 (− 17.6 to − 1.8)	0.02*	− 7.1 (− 17.4 to 3.2)	0.18
Hyperactivity	29.4 (21.1 to 37.8)	27.7 (19.0 to 36.4)	28.0 (20.1 to 35.8)	− 1.7 (− 4.5 to 1.1)	0.24	− 1.5 (− 5.0 to 2.0)	0.41
Irritability	25.0 (12.9 to 37.1)	22.2 (9.3 to 35.2)	22.8 (10.1 to 35.5)	− 2.8 (− 6.1 to 0.6)	0.11	− 2.2 (− 5.8 to 1.3)	0.22
Inappropriateness	5.3 (−0.4 to 10.9)	5.0 (− 0.8 to 10.8)	5.0 (−1.0 to 11.1)	− 0.3 (− 0.8 to 0.3)	0.35	− 0.2 (− 1.3 to 0.9)	0.72
Social withdrawal	34.3 (22.8 to 45.7)	30.7 (20.3 to 41.0)	31.2 (19.8 to 42.7)	− 3.6 (− 5.8 to − 1.4)	0.001*	− 3.0 (− 5.6 to − 0.4)	0.02*
Stereotypy	9.9 (6.5 to 13.4)	8.4 (4.9 to 11.8)	9.8 (6.5 to 13.1)	− 1.5 (− 2.8 to − 0.3)	0.02*	− 0.09 (− 1.8 to 1.6)	0.92
Social responsiveness scale total score	154.1 (106.6 to 201.7)	147.0 (99.9 to 194.1)	144.4 (95.5 to 193.3)	− 7.1 (− 16.9 to 2.6)	0.15	−9.7 (− 18.7 to − 0.8)	0.03*
Awareness	22.2 (15.7 to 28.7)	22.5 (16.2 to 28.7)	22.7 (15.8 to 29.5)	0.3 (− 0.9 to 1.4)	0.65	0.5 (− 1.2 to 2.1)	0.60
Cognition	26.6 (15.3 to 37.9)	25.4 (12.8 to 38.0)	25.1 (14.0 to 36.3)	− 1.2 (− 4.2 to 1.9)	0.44	− 1.5 (− 4.1 to 1.1)	0.27
Communication	55.6 (35.3 to 75.9)	52.7 (32.8 to 72.7)	50.6 (30.5 to 70.8)	− 2.9 (− 6.5 to 0.8)	0.13	− 5.0 (− 8.4 to −1.5)	0.005*
Mannerisms	24.1 (19.1 to 29.1)	22.7 (16.7 to 28.7)	22.8 (17.3 to 28.3)	− 1.5 (− 3.3 to 0.4)	0.12	− 1.3 (− 3.7 to 1.1)	0.28
Motivation	27.1 (17.5 to 36.8)	24.6 (15.5 to 33.6)	24.0 (13.6 to 34.3)	− 2.6 (− 4.2 to − 0.9)	0.003*	− 3.1 (− 5.1 to − 1.2)	0.001*

Mean change scores were adjusted for age and gender

*Statistically significant change from baseline

### Changes in symptoms

The change in ABC and SRS scores over the study period are shown in Table 2. Mean scores on both measures showed improvements (decreases) over the 3-month study period, but only the change in the SRS was significant. The ABC improved − 7.1 points (95% CI − 17.4 to 3.2), and the SRS improved − 9.7 points (95% CI − 18.7 to − 0.8).

We also examined the number of participants who had a “clinical response,” defined as an improvement in four or more points in the ABC. Eight participants had a clinical response compared to seven who were classified as non-responders. Responders exhibited a 21.8-point decrease (improvement) in total ABC (*p* <  0.001) and a 20.2-point decrease in SRS (*p* <  0.001), compared to increases of 10 points in ABC (*p* = 0.001) and 8 points in SRS (*p* = 0.076) for non-responders.

**Fig. 1:**
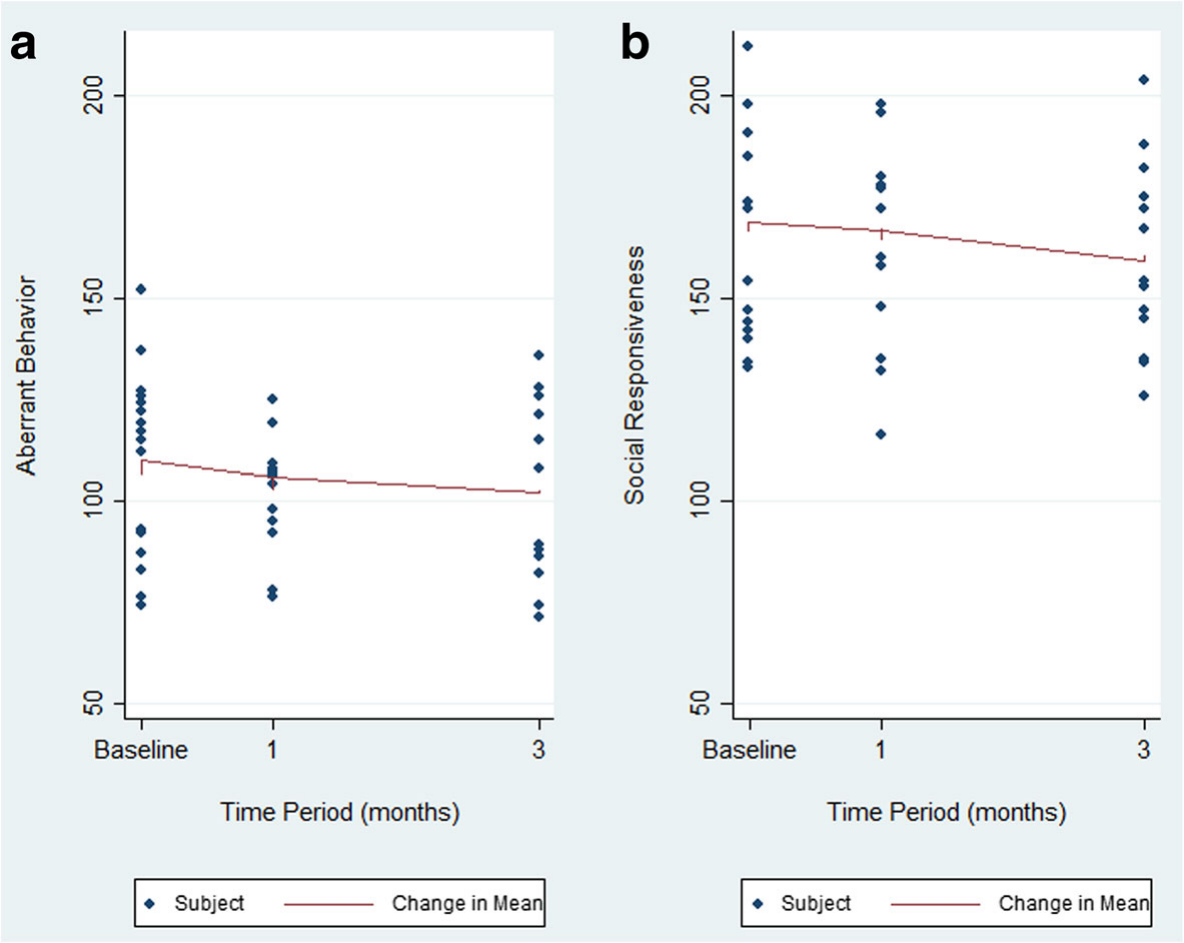
Change in mean outcome scores over time. **a** Change in mean aberrant behavior (ABC). **b** Change in mean social responsiveness (SRS). Mean scores were adjusted for sex and age of subjects. Decreasing score indicates clinical improvement

Figure 1 demonstrates the marked improvement at 1-month for the ABC and a more gradual improvement from 1 to 3 months. The SRS demonstrates a slower initial improvement at 1 month with continued improvement to a significant change at 3 months.

Parents were asked to provide subjective descriptions of the changes they noted in their children, and seven parents provided responses (Table 3).

**Table 3:** Parental descriptions of behavior change

Subject	Parental description
1	Increased conversation, asks questions, makes jokes, “more with us,” decreased vocalizations
2	Engaging, more lucid, relaxed
3	More engaged, increased eye contact, more attentive, calmer
4	More flexibility
5	Less repetitive behavior, calmer, more language at school
6	Energy burst after taking supplement, difficult behavior if misses a dose, eager to try things, meltdowns improved (fewer, easier to “get out of,” shorter duration)
7	Estimated 85% decrease in duration, frequency, and intensity of self-injurious behavior

### Correlations between metabolite changes and clinical changes

The metabolite analysis measured 694 different urinary metabolites. Table 4 shows all urinary metabolites with correlations with an absolute value ≥ 0.6 with either the ABC or the SRS.

**Table 4:** Metabolite correlations

	Primary outcome measure				
Metabolite	ABC Corr. *p* value		SRS Corr. *p* value		Metabolic pathway
Amino acids (endogenous)					
Arginine	0.63	0.03			Amino acid
Assymetric dimethylarginine (ADMA)			− 0.61	0.06	Arginine catabolite (endogenous inhibitor of nitric oxide synthases)
*N*-delta-acetylornithine			0.62	0.06	Arginine catabolite
*N*-acetylputrescine			− 0.65	0.04	Arginine catabolite
4-Acetamidobutanoate			− 0.62	0.06	Arginine catabolite
Tryptophan	− 0.67	0.02			Amino acid
Tyrosine	− 0.67	0.02			Amino acid
Theanine			0.7	0.02	a.a. deriv. from tea—of food origin
Carnosine			0.6	0.07	a.a. deriv.; dietary; antioxidant; CNS functionality
β-hydroxyisovalerate	− 0.73	0.007			a.a. metabolism (leucine)
α-hydroxyisocaproate	− 0.66	0.02			a.a. metabolite (leucine)
4-Methyl-2-oxopentanoate			− 0.62	0.06	a.a. metabolite (leucine)
α-hydroxyisovalerate	− 0.6	0.04			a.a. metabolism (isoleucine)
2-Methylbutyrylcarnitine (C5)			0.6	0.07	a.a. derived (isoleucine)
3-Hydroxyisobutyrate			− 0.72	0.02	a.a. metabolism (valine)
*N*-carbamoylalanine	0.73	0.01			a.a. metabolite (alanine)
*N*-methyltaurine			− 0.63	0.05	a.a. metabolite (cysteine)
Taurine	− 0.6	0.04			a.a. metabolite (cysteine); critical for oxidative stress
Dimethylglycine			− 0.61	0.06	a.a. metabolite (glycine)
1-Methylguanidine			0.7	0.02	a.a. metabolism
*N*-acetylhistamine	0.69	0.01			a.a. metabolite (histidine)
Amino acids (microbiome-associated or contributed)					
Tyramine O-sulfate	0.61	0.03			a.a. metabolite (tyrosine); catecholamine trigger
3-Indoxyl sulfate	0.63	0.03			a.a. metabolite (tryptophan); likely of gut microbiome origin
2-Oxindole-3-acetate			0.61	0.06	a.a. metabolite (tryptophan); likely of gut microbiome origin
Indolin-2-one	0.61	0.03			a.a. metabolite (tryptophan); likely of gut microbiome origin
Phenyllactate (PLA)			− 0.61	0.06	a.a. metabolite (phenylalanine), contribution from gut microbiome
Phenylacetylglutamine	0.65	0.02			a.a. metabolite (glutamine); likely of gut microbiome origin
Tryptophan betaine			− 0.69	0.03	a.a. (contribution from microbiome)
*N*-acetyl-cadaverine	0.65	0.02			a.a. degradation product (lysine); likely of gut microbiome origin
Benzene metabolism					
Hydroquinone sulfate			− 0.66	0.04	Benzene metabolite; likely of exogenous origin
Caffeic acid derivatives					
Chlorogenate			0.61	0.06	Caffeic acid deriv.; dietary/food additive
Cholesterol metabolism					
Cholesterol	− 0.75	0.005			Cholesterol
Cholate			− 0.73	0.02	Cholesterol (bile acid)
12-Dehydrocholate			− 0.69	0.03	Cholesterol (bile acid), microbiome origin
7-Ketodeoxycholate			− 0.6	0.07	Cholesterol (bile acid), microbiome origin
Glycocholenate sulfate	− 0.86	< 0.001			Cholesterol (bile acid), microbiome origin
Cortisone	− 0.8	0.002			Cholesterol (hormone)
Cortisol 21-glucuronide	− 0.73	0.008			Cholesterol (hormone)
Epiandrosterone glucuronide	− 0.68	0.01			Cholesterol (hormone)
17α-hydroxypregnanolone glucuronide	− 0.61	0.03			Cholesterol (hormone)
5α-androstan-3β,17α-diol disulfate	− 0.61	0.04			Cholesterol (hormone)
Pregnen-diol disulfate			− 0.82	0.004	Cholesterol (hormone)
Dehydroepiandrosterone glucuronide			− 0.78	0.008	Cholesterol (hormone)
11-Ketoetiocholanolone sulfate			− 0.76	0.01	Cholesterol (hormone)
5α-pregnan-3β,20α-diol disulfate			− 0.69	0.03	Cholesterol (hormone)
5α-pregnan-3(α/β),20β-diol disulfate			− 0.68	0.03	Cholesterol (hormone)
21-Hydroxypregnenolone disulfate			− 0.65	0.04	Cholesterol (hormone)
3α,21-dihydroxy-5β-pregnane-11,20-dione 21-glucuronide			− 0.61	0.06	Cholesterol (hormone)
Androstenediol 3β 17β d0			− 0.6	0.07	Cholesterol (hormone)
Fatty acids					
Diglycerol	0.66	0.02			Lipid, likely of exogenous origin
Pimelate (heptanedioate)	0.61	0.03	0.61	0.06	Dicarboxylate fatty acid (DFA)
Suberate (octanedioate)			0.75	0.01	Dicarboxylate fatty acid (DFA)
Azeloylcarnitine (C9-DC)	0.66	0.02			Acylcarnitine-conjugated DFA
Microbial polycyclic aromatic hydrocarbon (PAH) degradation					
Gentisate (2,5-diOH-benzoate)			0.69	0.03	Key intermediate in microbial PAH degradation
4-Hydroxybenzoate	− 0.63	0.03			Gut microbiome/amino acid
3-Ethylphenylsulfate	− 0.63	0.03			Gut microbiome/amino acid
Monoterpene phenol					
Thymol sulfate	0.62	0.03			Monoterpene phenol of food origin
Neurotransmitters					
*N*-methylglutamate			0.65	0.04	Neurotransmitter
Glutamine	− 0.77	0.003			Neurotransmitter
Homovanillate (HVA)	− 0.62	0.03	− 0.65	0.04	Neurotransmitter
Hypoxanthine	− 0.62	0.03	− 0.64	0.05	Neurotransmitter
Serotonin	−0.61	0.03			Neurotransmitter
Oxidative stress					
γ-glutamylglutamine	− 0.63	0.03			Oxidative stress
Methionine sulfone	− 0.62	0.03			Oxidative stress
Polyol					
3-Carboxy-4-methyl-5-propyl-2-furanpropanoate (CMPF)			− 0.78	0.008	Polyol associated with uremia
Sphingomyelin					
Palmitoyl sphingomyelin (d18:1/16:0)	− 0.69	0.01			Sphingomyelin
Stearoyl sphingomyelin (d18:1/18:0)	− 0.77	0.004			Sphingomyelin
Lignoceroyl sphingomyelin (d18:1/24:0)	− 0.74	0.006			Sphingomyelin
Behenoyl sphingomyelin (d18:1/22:0)	− 0.74	0.006			Sphingomyelin
Sphingomyelin (d18:1/20:1, d18:2/20:0)	− 0.76	0.004			Sphingomyelin
Sphingomyelin (d18:1/14:0, d16:1/16:0)	− 0.75	0.005			Sphingomyelin
Sphingomyelin (d18:1/20:0, d16:1/22:0)	− 0.74	0.006			Sphingomyelin
Sugars					
Arabinose			− 0.7	0.02	Sugar frequently associated with intestinal *Candida* overgrowth
TCA cycle					
Malate	− 0.65	0.02			TCA cycle intermediate
Other					
Gentisic acid-5-glucoside			0.65	0.04	Xenobiotic (chemical)
Erythritol			− 0.83	0.003	Xenobiotic (diet-derived)
1-Palmitoyl-2-oleoyl-GPE (16:0/18:1)	− 0.72	0.008			Phospholipid

*a.a* amino acid, *PAH* polycyclic aromatic hydrocarbon

Positive correlations indicate that, as the urinary metabolite increased, the ABC and SRS also increased (worsened). Negative correlations indicate that, as the urinary metabolite increased, the ABC and SRS decreased (improved). Metabolites that fall into similar, known metabolic pathways are highlighted and an interpretation is provided in the discussion.

### Adverse events

Six families reported that their child had a new medical event during the study period: one nausea and vomiting (but also associated with pica), one stomach flu, one inflammation in the esophagus and weight gain, one ruptured appendix, and one weight loss. The one serious adverse event (ruptured appendix) required hospitalization and surgery but led to no long-term complications. The committee on human research review determined that it was unlikely to be related to the study supplement.

## Discussion

This study builds upon findings from one prior randomized, placebo-controlled trial, which found that the use of sulforaphane led to improvements in behavior and social responsiveness in children and young adults (aged 13–27) with ASD [19]. Our primary goal was to examine changes in metabolites in children with ASD who were taking sulforaphane to determine a possible mechanism of action. We observed that a group of school-age children (mean age 14.8) showed a trend towards improvement in behavior (ABC) and a statistically significant improvement in social responsiveness after 12 weeks of treatment. The magnitude of improvements in the current study (− 9.7 points for the SRS and − 7.1 points for the ABC) were smaller than in the prior study (− 20.4 points for the SRS and − 21.4 points for the ABC), which may be related to the younger age of these participants or other differences in the study populations. One important difference in the study populations is that in the current study, all of the children were attending one specialized school with programs designed for children with ASD. The behavioral interventions in place at the school may have already been producing positive effects (at baseline) and limited the ability to detect further improvement from sulforaphane over the 12-week study (when compared to the prior study, where subjects were not in the same school).

In order to determine which metabolites might mediate clinical improvements, we examined correlations between the change in urinary metabolite levels and change in behavior and social responsiveness over the 12-week study. We defined, a priori, that a correlation with an absolute value of 0.6 or greater was potentially relevant and might indicate a mechanism of action. Of 694 measured urinary metabolites, 77 had correlations with an absolute value of ≥ 0.6. While it is not possible to discuss the implications of all of these correlations, several of these metabolite changes cluster into known pathways (Table 4) that have been reported to be altered in children with ASD. These pathways involve metabolites involved in oxidative stress, amino acid/gut microbiome, neurotransmitters, hormones/stress response, and sphingomyelin metabolism, among others. Sulforaphane may affect these pathways through a variety of mechanisms including Nrf2-mediated induction of phase 2 detoxification enzymes, suppression of cytochrome P450 enzymes, induction of apoptotic pathways, and anti-inflammatory activity that have been described in detail [28].

Many prior studies have found increased oxidative stress in children with ASD, which may be due to increased production or decreased clearance of reactive oxygen species (also known as ROS, or oxygen free-radicals). Children with ASD have been found to have lower levels of the metabolites that process oxygen free radicals (methionine, *S*-adenosylmethionine, homocysteine, cystathionine, cysteine, and total glutathione), higher levels of metabolites that are involved in the body’s mechanism for reducing oxidative stress (oxidized glutathione, adenosine, and *S*-adenosylhomocysteine), and markers of protein and DNA oxidative damage [3, 29]. Also, two prior randomized controlled trials—one of the antioxidant NAC, and one of the methyl donor, methyl B12—have found that these treatments improve clinical symptoms in children with ASD [17, 18]. In the methyl B12 supplementation study, clinical improvements were correlated with increases in plasma methionine, decreases in *S*-adenosyl-homocysteine (SAH), and improvements in the ratio of *S*-adensylmethionine (SAM) to SAH [18]. Interestingly, in the current study, clinical improvements were correlated with two metabolites known to be involved in redox metabolism. The negative correlations found with γ-glutamylglutamine and methionine sulfone indicate that, as the urinary levels of these metabolites increased, the symptoms scores decreased (improved). This suggests that sulforaphane may mediate beneficial clinical effects through increases in antioxidant capacity, which is one of its well-documented physiological effects [20].

Abnormalities in amino acid metabolism have been reported in children with ASD compared to control children [9, 12, 14, 16], and this may be related to altered processing of amino acids by gut microbiota [30]. We found correlations between clinical improvement and the amino acids tryptophan, tyrosine, and assymetric-dimethylarginine (ADMA, a derivative of the amino acid, arginine). The involvement of altered amino acids in the pathology of ASD is plausible since amino acids are building blocks for many key neurotransmitters and hormones, including catecholamines and serotonin [30]. Six prior studies using urinary metabolomics noted increased urinary tryptophan in children with ASD [7, 9, 12, 14, 16, 30], and tryptophan is a key substrate in the serotonergic metabolic pathway. For tryptophan, tyrosine, and ADMA, the correlations in the current study were all negative, indicating that as the urinary levels of these amino acids increased, symptom scores decreased (improved). We also identified correlations between clinical improvement and changes in a number of other amino acids that are known to be associated with gut microbiota. Six of the eight metabolites in this category had positive correlations, indicating that decreased urinary levels were associated with improved clinical symptoms. It is not yet clear how sulforaphane may affect or improve the amino acid abnormalities (including those associated with the gut microbiome) and lead to clinical improvements.

We also found associations between clinical improvements and changes in five urinary neurotransmitter-related metabolites, including *N*-methylglutamate, glutamine, hypoxanthine, serotonin, and homovanillate (HMV), which is the normal end product of dopamine degradation and was elevated in the urine of children with ASD in a prior study [14]. For all of the urinary neurotransmitters except *N*-methylglutamate, the correlations were negative, indicating that increases in the urinary levels of these metabolites were associated with lower scores on the ABC and SRS and therefore improved symptoms. This suggests that either increased production or increased elimination of these metabolites is correlated with beneficial effects. Hypoxanthine, which is part of the purine pathway, was previously found to be elevated in children with ASD [9]. We also found that urinary glutamine was correlated with clinical improvements. Glutamatergic dysfunction has been hypothesized to be involved in the pathogenesis of ASD, with several studies reporting abnormal levels of glutamate in various regions in the brain [13].

We found correlations between clinical improvements and a large number of hormones, some of them stress-related. Prior studies have found higher salivary stress hormones in children with ASD and higher hair cortisol levels, suggesting both acute and chronic elevation in stress hormones [31, 32]. Higher hair cortisol levels were associated with more severe autism symptoms and anxiety [31]. In the current study, both cortisone and cortisol-21-glucuronide had negative correlations with ASD-related behavior, indicating that increased urinary levels were associated with improved symptoms. ASD behavior and social responsiveness were also related to a large number of other hormones, and all of these correlations were negative, again indicating that increased urinary levels of various hormones were associated with improved symptoms. It is not clear if this indicates that increased production or increased excretion is associated with improvement, but it highlights changes in hormonal function in ASD as an area for further study.

A novel finding of the current study is that improvement in behavior was correlated with seven different chemical forms of sphingomyelin. Seven sphingomyelin metabolites were each strongly negatively correlated with behavior, such that increased urinary levels were associated with improved behavior. Sphingomyelin is a sphingolipid found in animal cell membranes, especially in the membranous myelin sheath that surrounds nerve cells and axons. To our knowledge, there have been no prior reports of abnormalities in sphingolipid levels in children with ASD, but there are numerous studies documenting abnormalities in the size, number, and morphology of dendrites in autism, which is related to altered synapse function [33]. Furthermore, sphingomyelin abnormalities have been noted in a number of other central nervous system disorders, including depression, anxiety, Alzheimer’s disease, and amyotrophic lateral sclerosis, suggesting that it may have a central role in normal brain development and function [34–37]. It is not clear how sulforaphane might alter sphingomyelin metabolism or availability and whether this is related to clinical benefits, but if this association is confirmed, it has important clinical and treatment implications.

The current study has a number of limitations. Importantly, this was a pilot study to investigate whether metabolomics might be a useful tool to suggest pathways that may be involved in the mechanism of action of treatments (in this case, sulforaphane) in ASD. The study was open label and parent raters may have rated more positively knowing that their child was taking the sulforaphane, although the magnitude of benefit is lower than in the one prior randomized controlled trial. The ratings were also limited to the ABC and SRS to minimize respondent burden, and while these are widely used outcome tools in ASD, we did not include other measures of repetitive behavior or adaptive function, which might have shown clinical changes in other important areas. Many factors affect urinary metabolomics, including diet, environment, stress, sleep, age, and other factors. In the current study, the wide age range (7–21), different gender and pubertal state of subjects and the small sample size all limit the strength of the conclusions regarding urinary metabolomic changes. The variation in these environmental effects may have been minimized by having all the children attending the same school 5 days a week. However, the findings of this study should be viewed as hypothesis generating and should be confirmed in future studies with larger sample sizes. Future studies would also benefit from plasma biochemical assessments of antioxidant status pre- and post-treatment since this is likely a key pathway in the mechanism of action of sulforaphane. We found that change in the two outcome measures, ABC and SRS, were associated with mostly different metabolites. We believe this is to be expected since the measures assess different components of human behavior and are likely influenced by different metabolic pathways. Finally, we used a different delivery method of sulforaphane than in a prior randomized controlled trial in autism [19] by providing a precursor, glucoraphanin, along with a conversion enzyme, myrosinase. Although the dosing was designed to produce a similar level of sulforaphane, it is possible that differences in bioavailable sulforaphane levels between the two studies could have led to differences in clinical results.

## Conclusions

We are not aware of any prior studies that have examined changes in urinary metabolites during a clinical trial of a treatment for autism. In this pilot study, we demonstrated the feasibility of using metabolomics to identify urinary metabolites that are correlated with clinical improvements and might therefore represent a mechanism of action. One group of metabolites in particular, the sphingolipid/sphingomyelin group, was highlighted as being significantly associated with improvement. Other urinary metabolite changes that were correlated with clinical improvement are related to oxidative stress, amino acid metabolism/gut microbiome metabolites, neurotransmitters, stress, and other hormones. These findings suggest that urinary metabolomics may be a tool to identify important changes in the quest to determine how certain biological interventions work to reduce specific symptoms of ASD. This may further provide clues to the underlying, active pathophysiology of ASD and may allow for more precise interventions targeted to the unique metabolome of each individual with this disorder.

## Abbreviations

ABC: Aberrant Behavior Checklist
ADMA: Assymetric dimethylarginine
ASD: Autism spectrum disorder
HMV: Homovanillate
NAC: *N*-acetylcysteine
SAH: *S*-adenosyl-homocysteine
SAM: *S*-adensylmethionine
SRS: Social Responsiveness Scale
UCSF: University of California, San Francisco

## References

[1] Rossignol DA, Frye RE (2012). A review of research trends in physiological abnormalities in autism spectrum disorders: immune dysregulation, inflammation, oxidative stress, mitochondrial dysfunction and environmental toxicant exposures. Mol Psychiatry.

[2] Ashwood P, Wills S, Van de Water J (2006). The immune response in autism: a new frontier for autism research. J Leukoc Biol.

[3] James SJ, Cutler P, Melnyk S, Jernigan S, Janak L, Gaylor DW, Neubrander JA (2004). Metabolic biomarkers of increased oxidative stress and impaired methylation capacity in children with autism. Am J Clin Nutr.

[4] Rossignol DA, Bradstreet JJ (2008). Evidence of mitochondrial dysfunction in autism and implications for treatment. Am J Biochem Biotechnol.

[5] Liu H, Talalay P, Fahey JW (2016). Biomarker-guided strategy for treatment of autism spectrum disorder (ASD). CNS Neurol Disord Drug Targets.

[6] National Research Council. Educating Children with Autism. Committee on Educational Interventions for Children with Autism. Catherine Lord and James P. McGee, eds. Division of Behavioral and Social Sciences and Education. Washington, DC: National Academy Press; 2001.

[7] Dieme B, Mavel S, Blasco H, Tripi G, Bonnet-Brilhault F, Malvy J, Bocca C, Andres CR, Nadal-Desbarats L, Emond P (2015). Metabolomics study of urine in autism spectrum disorders using a multiplatform analytical methodology. J Proteome Res.

[8] Emond P, Mavel S, Aidoud N, Nadal-Desbarats L, Montigny F, Bonnet-Brilhault F, Barthelemy C, Merten M, Sarda P, Laumonnier F (2013). GC-MS-based urine metabolic profiling of autism spectrum disorders. Anal Bioanal Chem.

[9] Gevi F, Zolla L, Gabriele S, Persico AM (2016). Urinary metabolomics of young Italian autistic children supports abnormal tryptophan and purine metabolism. Mol Autism.

[10] Kuwabara H, Yamasue H, Koike S, Inoue H, Kawakubo Y, Kuroda M, Takano Y, Iwashiro N, Natsubori T, Aoki Y (2013). Altered metabolites in the plasma of autism spectrum disorder: a capillary electrophoresis time-of-flight mass spectroscopy study. PLoS One.

[11] Mavel S, Nadal-Desbarats L, Blasco H, Bonnet-Brilhault F, Barthelemy C, Montigny F, Sarda P, Laumonnier F, Vourc’h P, Andres CR (2013). 1H-13C NMR-based urine metabolic profiling in autism spectrum disorders. Talanta.

[12] Ming X, Stein TP, Barnes V, Rhodes N, Guo L (2012). Metabolic perturbance in autism spectrum disorders: a metabolomics study. J Proteome Res.

[13] Nadal-Desbarats L, Aidoud N, Emond P, Blasco H, Filipiak I, Sarda P, Bonnet-Brilhault F, Mavel S, Andres CR (2014). Combined 1H-NMR and 1H-13C HSQC-NMR to improve urinary screening in autism spectrum disorders. Analyst.

[14] Noto A, Fanos V, Barberini L, Grapov D, Fattuoni C, Zaffanello M, Casanova A, Fenu G, De Giacomo A, De Angelis M (2014). The urinary metabolomics profile of an Italian autistic children population and their unaffected siblings. J Matern Fetal Neonatal Med.

[15] West PR, Amaral DG, Bais P, Smith AM, Egnash LA, Ross ME, Palmer JA, Fontaine BR, Conard KR, Corbett BA (2014). Metabolomics as a tool for discovery of biomarkers of autism spectrum disorder in the blood plasma of children. PLoS One.

[16] Yap IK, Angley M, Veselkov KA, Holmes E, Lindon JC, Nicholson JK (2010). Urinary metabolic phenotyping differentiates children with autism from their unaffected siblings and age-matched controls. J Proteome Res.

[17] Hardan AY, Fung LK, Libove RA, Obukhanych TV, Nair S, Herzenberg LA, Frazier TW, Tirouvanziam R (2012). A randomized controlled pilot trial of oral N-acetylcysteine in children with autism. Biol Psychiatry.

[18] Hendren RL, James SJ, Widjaja F, Lawton B, Rosenblatt A, Bent S (2016). Randomized, placebo-controlled trial of methyl B12 for children with autism. J Child Adolesc Psychopharmacol.

[19] Singh K, Connors SL, Macklin EA, Smith KD, Fahey JW, Talalay P, Zimmerman AW (2014). Sulforaphane treatment of autism spectrum disorder (ASD). Proc Natl Acad Sci U S A.

[20] Fahey JW, Talalay P (1999). Antioxidant functions of sulforaphane: a potent inducer of phase II detoxication enzymes. Food Chem Toxicol.

[21] Fahey JW, Holtzclaw WD, Wehage SL, Wade KL, Stephenson KK, Talalay P (2015). Sulforaphane bioavailability from glucoraphanin-rich broccoli: control by active endogenous myrosinase. PLoS One.

[22] Long T, Hicks M, Yu HC, Biggs WH, Kirkness EF, Menni C, Zierer J, Small KS, Mangino M, Messier H (2017). Whole-genome sequencing identifies common-to-rare variants associated with human blood metabolites. Nat Genet.

[23] Dehaven CD, Evans AM, Dai H, Lawton KA (2010). Organization of GC/MS and LC/MS metabolomics data into chemical libraries. J Cheminform.

[24] Chetwynd AJ, Abdul-Sada A, Holt SG, Hill EM (2016). Use of a pre-analysis osmolality normalisation method to correct for variable urine concentrations and for improved metabolomic analyses. J Chromatogr A.

[25] Rothman KJ (1990). No adjustments are needed for multiple comparisons. Epidemiology.

[26] Mukaka MM (2012). Statistics corner: a guide to appropriate use of correlation coefficient in medical research. Malawi Med J.

[27] McCracken JT, McGough J, Shah B, Cronin P, Hong D, Aman MG, Arnold LE, Lindsay R, Nash P, Hollway J (2002). Risperidone in children with autism and serious behavioral problems. N Engl J Med.

[28] Juge N, Mithen RF, Traka M (2007). Molecular basis for chemoprevention by sulforaphane: a comprehensive review. Cell Mol Life Sci.

[29] Melnyk S, Fuchs GJ, Schulz E, Lopez M, Kahler SG, Fussell JJ, Bellando J, Pavliv O, Rose S, Seidel L (2012). Metabolic imbalance associated with methylation dysregulation and oxidative damage in children with autism. J Autism Dev Disord.

[30] Lussu M, Noto A, Masili A, Rinaldi AC, Dessi A, De Angelis M, De Giacomo A, Fanos V, Atzori L, Francavilla R. The urinary 1 H-NMR metabolomics profile of an Italian autistic children population and their unaffected siblings. Autism Res. 2017;10(6):1058–66. 10.1002/aur.1748 28296209

[31] Ogawa S, Lee YA, Yamaguchi Y, Shibata Y, Goto Y (2017). Associations of acute and chronic stress hormones with cognitive functions in autism spectrum disorder. Neuroscience.

[32] Putnam SK, Lopata C, Thomeer ML, Voker MA, Rodgers JD (2015). Salivary cortisol levels and diurnal patterns in children with autism spectrum disorder. J Dev Physic Disabil.

[33] Martinez-Cerdeno V (2017). Dendrite and spine modifications in autism and related neurodevelopmental disorders in patients and animal models. Dev Neurobiol.

[34] Berkecz R, Tomosi F, Kormoczi T, Szegedi V, Horvath J, Janaky T (2017). Comprehensive phospholipid and sphingomyelin profiling of different brain regions in mouse model of anxiety disorder using online two-dimensional (HILIC/RP)-LC/MS method. J Pharm Biomed Anal.

[35] Blasco H, Veyrat-Durebex C, Bocca C, Patin F, Vourc’h P, Kouassi Nzoughet J, Lenaers G, Andres CR, Simard G, Corcia P (2017). Lipidomics reveals cerebrospinal-fluid signatures of ALS. Sci Rep.

[36] Dinoff A, Saleem M, Herrmann N, Mielke MM, Oh PI, Venkata SLV, Haughey NJ, Lanctot KL (2017). Plasma sphingolipids and depressive symptoms in coronary artery disease. Brain and behavior.

[37] Kosicek M, Zetterberg H, Andreasen N, Peter-Katalinic J, Hecimovic S (2012). Elevated cerebrospinal fluid sphingomyelin levels in prodromal Alzheimer’s disease. Neurosci Lett.

